# Whole-Genome Sequencing-Based Characterization of 100 Listeria monocytogenes Isolates Collected from Food Processing Environments over a Four-Year Period

**DOI:** 10.1128/mSphere.00252-19

**Published:** 2019-08-07

**Authors:** Daniel Hurley, Laura Luque-Sastre, Craig T. Parker, Steven Huynh, Athmanya K. Eshwar, Scott V. Nguyen, Nicholas Andrews, Alexandra Moura, Edward M. Fox, Kieran Jordan, Angelika Lehner, Roger Stephan, Séamus Fanning

**Affiliations:** a UCD-Centre for Food Safety, School of Public Health, Physiotherapy and Sports Science, University College Dublin, Dublin, Ireland; b School of Agriculture and Food Science, University College Dublin, Dublin, Ireland; c Western Regional Research Center, Produce Safety and Microbiology Research Unit, Agricultural Research Service, U.S. Department of Agriculture, Albany, California, USA; d Institute for Food Safety and Hygiene, University of Zurich, Zurich, Switzerland; e Biodiversity and Epidemiology of Bacterial Pathogens, Institut Pasteur, Paris, France; f Department of Applied Sciences, Northumbria University, Newcastle upon Tyne, United Kingdom; g Food Safety Department, Teagasc Food Research Centre, Fermoy, County Cork, Ireland; University of Kentucky

**Keywords:** *Listeria monocytogenes*, foodborne pathogens, persistence, virulence

## Abstract

Listeria monocytogenes is frequently found in foods and processing facilities, where it can persist, creating concerns for the food industry. Its ability to survive under a wide range of environmental conditions enhances the potential for cross-contamination of the final food products, leading to possible outbreaks of listeriosis. In this study, whole-genome sequencing (WGS) was applied as a tool to characterize and track 100 L. monocytogenes isolates collected from three food processing environments. These WGS data from environmental and food isolates were analyzed to (i) assess the genomic diversity of L. monocytogenes, (ii) identify possible source(s) of contamination, cross-contamination routes, and persistence, (iii) detect absence/presence of antimicrobial resistance-encoding genes, (iv) assess virulence genotypes, and (v) explore *in vivo* pathogenicity of selected L. monocytogenes isolates carrying different virulence genotypes. The predominant L. monocytogenes sublineages (SLs) identified were SL101 (21%), SL9 (17%), SL121 (12%), and SL5 (12%). Benzalkonium chloride (BC) tolerance-encoding genes were found in 62% of these isolates, a value that increased to 73% among putative persistent subgroups. The most prevalent gene was *emrC* followed by *bcrABC*, *qacH*-Tn*6188*, and *qacC.* The L. monocytogenes major virulence factor *inlA* was truncated in 31% of the isolates, and only one environmental isolate (L. monocytogenes CFS086) harbored all major virulence factors, including *Listeria* pathogenicity island 4 (LIPI-4), which has been shown to confer hypervirulence. A zebrafish embryo infection model showed a low (3%) embryo survival rate for all putatively hypervirulent L. monocytogenes isolates assayed. Higher embryo survival rates were observed following infection with unknown virulence potential (20%) and putatively hypovirulent (53 to 83%) L. monocytogenes isolates showing predicted pathogenic phenotypes inferred from virulence genotypes.

**IMPORTANCE** This study extends current understanding of the genetic diversity among L. monocytogenes from various food products and food processing environments. Application of WGS-based strategies facilitated tracking of this pathogen of importance to human health along the production chain while providing insights into the pathogenic potential for some of the L. monocytogenes isolates recovered. These analyses enabled the grouping of selected isolates into three putative virulence categories according to their genotypes along with informing selection for phenotypic assessment of their pathogenicity using the zebrafish embryo infection model. It has also facilitated the identification of those isolates with genes conferring tolerance to commercially used biocides. Findings from this study highlight the potential for the application of WGS as a proactive tool to support food safety controls as applied to L. monocytogenes.

## Abstract (precis)

This study extends current understanding of the genetic diversity among L. monocytogenes from various food products and food processing environments. Application of WGS-based strategies facilitated tracking of this pathogen of importance to human health along the production chain while providing insights into the pathogenic potential for some of the L. monocytogenes isolates recovered. These analyses enabled the grouping of selected isolates into three putative virulence categories according to their genotypes along with informing selection for phenotypic assessment of their pathogenicity using the zebrafish embryo infection model. It has also facilitated the identification of those isolates with genes conferring tolerance to commercially used biocides. Findings from this study highlight the potential for the application of WGS as a proactive tool to support food safety controls as applied to L. monocytogenes.

## INTRODUCTION

Listeria monocytogenes is an opportunistic foodborne pathogen with the third highest mortality rate among all bacterial foodborne pathogens in the United States (1). Many cases of human listeriosis arise following consumption of contaminated ready-to-eat foods (2). Listeriosis outbreaks represent an annual economic burden of $2.8 billion in the United States (3). In Europe, although the incidence of listeriosis is low, the European Food Safety Authority (EFSA) reported 2,206 confirmed human cases among the 28 European member states in 2015 (2). The annual number of cases has increased significantly in Ireland and the European Union (EU) since 2008 (4). For this reason, surveillance programs in food processing facilities can support food safety measures by expediting the detection, monitoring, and characterization of any persistent or sporadic isolates cultured from the production environment as well as the final product (5–8).

L. monocytogenes is ubiquitously found in the natural environment and has the ability to persist in food processing facilities for months and even years, despite the application of sanitation measures (5, 9, 10). The control of L. monocytogenes in the food processing industry is essential to reduce risk and protect the consumer (11). Food business operators must have an effective control strategy to minimize the harborage and potential dissemination of L. monocytogenes within their domain of responsibility. This includes workflow auditing, effective use of sanitizers, an environmental monitoring program, as well as an assessment of microbial quality for incoming ingredients (12). Any failure in one or more of these strategies can undermine the integrity of the safety program and compromise the safe production of food (11, 13). Persistence of L. monocytogenes in food processing facilities often results in the cross-contamination of the final product, increasing the risk of an outbreak (10, 14–16).

Clinical signs of listeriosis manifest in a wide variety of forms from mild gastroenteritis to a severe systemic infection characterized by septicemia and invasion of the central nervous system (CNS). During pregnancy, there is a risk of transplacental transmission leading to maternal-neonatal (MN) infection (17, 18). Although food regulatory authorities consider L. monocytogenes isolates to be equally pathogenic, it has been demonstrated that certain serotypes and clonal complexes (CCs) are more commonly encountered in clinical cases (19, 20). L. monocytogenes can be classified into four distinct evolutionary lineages (denoted I to IV) with most isolates grouping into lineages I and II (21, 22). Three serotypes in particular, 1/2a (lineage II), which is predominantly isolated from foods, along with serotypes 1/2b and 4b (both lineage I), are responsible for 95% of human cases reported, with 4b being the predominant serotype among clinical isolates and outbreaks (23).

L. monocytogenes CCs are typically divided into the following: (i) infection-associated isolates, which belong to lineage I and are strongly linked with clinical origins (including CC1, CC2, CC4, and CC6), (ii) food-associated isolates, which belong to lineage II and are predominantly within the production environment (including CC9 and CC121), and (iii) intermediate-associated isolates that are isolated from both clinical and food settings (19, 20). Infection-associated isolates, which may harbor *Listeria* pathogenicity island 4 (LIPI-4), are typically considered hypervirulent, as they represent a bigger threat to public health. LIPI-4 carries six genes annotated as cellobiose-type phosphotransferase systems that can enhance invasion, leading to CNS and MN listeriosis (19). In contrast, isolates with reduced pathogenicity can display premature stop codons (PMSCs) in one or more virulence factors, such as *actA*, *inlAB*, and the transcriptional regulator *prfA*, leading to truncated and potentially nonfunctional proteins (19, 24–26).

Epidemiological surveillance of L. monocytogenes has been traditionally performed using conventional molecular subtyping techniques, including pulsed-field gel electrophoresis (PFGE), multilocus variable-number tandem-repeat analysis (MLVA), and multilocus sequence typing (MLST) (27–29). These methods provide useful but lower-resolution information that cannot reliably distinguish hypervirulent isolates. In contrast, whole-genome sequencing (WGS) is increasingly being used as the primary epidemiological surveillance tool in national programs, outbreak investigations, and the environmental monitoring programs of food processing facilities to support food safety controls and protect public health (30–33).

In this study, surveillance of L. monocytogenes in three food processing environments was conducted over 4 years using WGS and bioinformatic analyses. This approach allowed for (i) assessment of the genomic diversity of L. monocytogenes, (ii) identification of potential sources of contamination, cross-contamination routes, and persistence, (iii) determination of the absence or presence of antimicrobial resistance-encoding genes, (iv) assessment of the virulence genotypes of the isolates recovered, and (v) prediction of the potential *in vivo* pathogenicity of L. monocytogenes isolates with different virulence genotypes. This study reinforces the utility and power of WGS combined with bioinformatic analyses and facilitated an investigation of the potential *in vivo* pathogenicity of L. monocytogenes with different virulence genotypes using the zebrafish embryo infection model. In time, these data can be translated to provide for a refinement of the food processing facility’s risk characterization and corrective action strategy.

## RESULTS

### Distribution of L. monocytogenes sublineages by isolation source.

The core genome MLST (cgMLST) profile of 1,748 loci was determined for all L. monocytogenes isolates, and analyses classified these isolates into 18 different sublineages (SLs). The SL designations were determined from cgMLST results, agreeing with CC designations determined from the seven-gene MLST scheme (see Table S1 in the supplemental material). The BIGSdb-*Lm* platform (https://bigsdb.pasteur.fr/listeria) enables the cgMLST genotyping method, which defines cgMLST types (CTs) as groups of cgMLST profiles that differ by up to 7 allelic mismatches out of 1,748 loci and SLs as groups of cgMLST profiles that differ by up to 150 allelic mismatches out of 1,748 loci (34, 35).

10.1128/mSphere.00252-19.1 TABLE S1 Bacterial strains used in this study. Download Table S1, XLSX file, 0.01 MB. Copyright © 2019 Hurley et al. 2019 Hurley et al. This content is distributed under the terms of the Creative Commons Attribution 4.0 International license.

**FIG 1:**
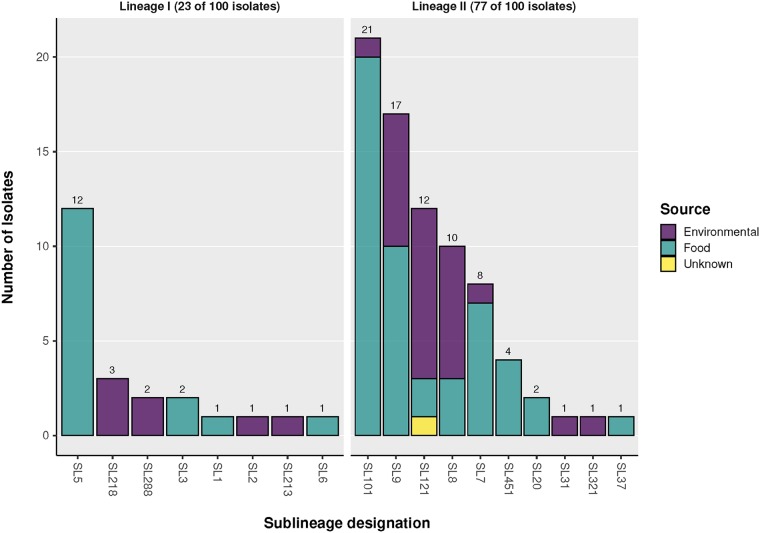
Sublineage distribution and source of Listeria monocytogenes isolates. The prevalence of cgMLST sublineages according to their source and evolutionary lineage are shown.

The predominant SLs identified included SL101 (21%), SL9 (17%), SL5 (12%), and SL121 (12%) (Fig. 1). SLs recovered from food were SL5, SL7, SL9, SL101, and SL451. Both SL8 and SL121 were identified in the food processing environments studied (Fig. 1). Certain SLs were uniquely recovered from food (including SL1, SL3, SL5, SL6, SL20, SL37, and SL451), whereas others were exclusively isolated from the environment (including SL2, SL31, SL213, SL218, SL288, and SL321) (Fig. 1). Isolates from clinically associated SLs (SL1, SL2, and SL6) were also identified among this collection with lower incidences relative to the incidences of the rest of the study isolates (Fig. 1). Furthermore, a new SL213 was identified and has been contributed to the BIGSdb-*Lm* platform (34).

### Identification of putative persistent strains using cgMLST.

**FIG 2:**
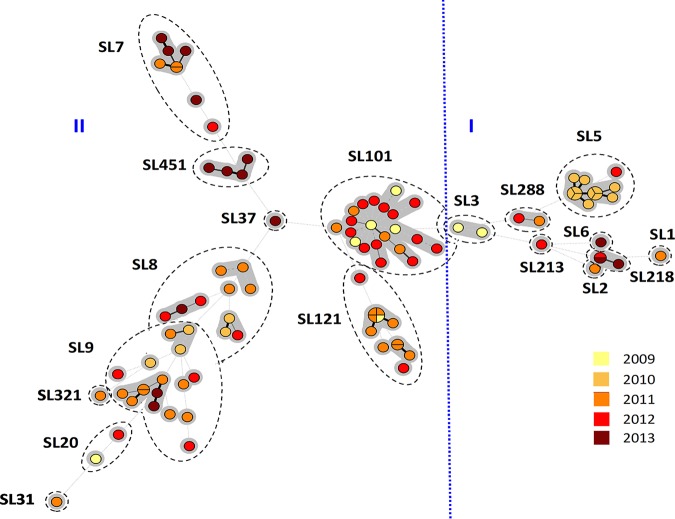
Minimum spanning tree based on the L. monocytogenes cgMLST profiles. cgMLST profiles are represented by circles, and the size of the circle is proportional to the number of isolates that share an identical cgMLST profile. Each circle is color coded by the year of isolation, and the length of lines connecting the cgMLST profiles is proportional to the number of allelic differences between circles. Dashed lines represent seven or more allelic differences between cgMLST profiles. A grey zone surrounds the group of circles that share the same cgMLST type (CT). SLs are indicated by dashed**-**line shapes. The vertical blue dashed line delineates lineage I and lineage II isolates.

**FIG 3:**
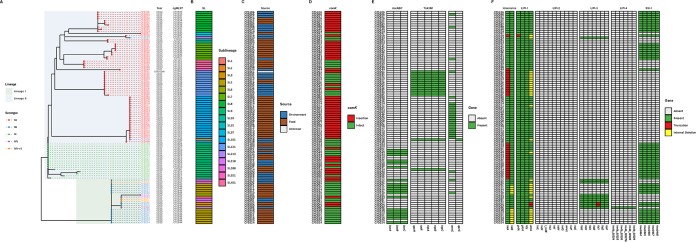
Phylogenetic distribution of study isolates and assessment of the *comK* region, benzalkonium chloride-encoding genotypes, and virulence factor genotypes across different sublineages. (A) The maximum likelihood phylogenetic tree based on a SNP matrix is color coded according to the serogroup as determined *in silico* from the WGS data of the isolates at the tip. The evolutionary lineage is highlighted in light green (lineage I) and blue (lineage II). (B) The year of isolation, cgMLST, and SL are added from left to right, followed by the source (C) and the *comK* gene (D) shown as either intact (green tiles) or disrupted (red tiles). (E) The next three columns show those genes associated with resistance to BC (presence [green] or absence [white] of gene). *tnpABC*, *tetR*, and *qacH* are encoded within Tn*6188* (HG329628). *bcrABC* may be carried on a plasmid or on the chromosome. *emrC* is carried on plasmid pLMST6 (Hx2000053480), and *qacC* is carried on plasmid pK5 (KJ792090.1). (F) The heatmap depicts the presence (green) or absence (grey) of proteins involved in L. monocytogenes virulence. When mutations, such as premature stop codons and internal deletions, were identified, they were highlighted in red and yellow, respectively.

A total of 37 distinct cgMLST types were identified, the most abundant being CT1526 (*n *=* *20), followed by CT1844 (*n *=* *11), and CT1828 (*n *=* *7), which belong to SL101, SL5, and SL9, respectively (Fig. 2 and Fig. 3A and B). Specific CTs have been repeatedly isolated from both food and environment sources over the 4-year period, suggesting potential persistence (Fig. 3A). In this study, presumptive persistent isolates were defined as the same CT being recovered at least three times in the processing plants with a minimum of 1 year between the first isolation and the last isolation. Based on this definition, six CTs (CT1526, CT1828, CT1833, CT1834, CT1836, and CT1839) were found to presumptively persist within the food processing environments in this study. Of these, 73% harbored a prophage within the *comK* gene (Fig. 3D). A prophage insertion within *comK* has previously been implicated in biofilm formation, persistence in food processing facilities, and virulence (10, 36, 37).

### Antimicrobial resistance and stress tolerance islands.

The fosfomycin resistance-encoding gene *fosX* was the only antibiotic resistance-encoding gene identified among the study isolates. This gene was present in all 100 isolates with sequence identity at the nucleotide level ranging between 92 and 100% relative to the AL591981 reference sequence.

In contrast, 55% of the isolates harbored BC tolerance-encoding genes (Fig. 3E). The most frequent BC tolerance-encoding gene identified was an efflux pump denoted as *emrC*, which was present in 25% of the isolates studied (38), followed by the previously characterized *bcrABC* cassette (19%) (39, 40). A transporter, QacH, putatively associated with the export of BC and encoded by a gene on transposon Tn*6188*, was identified in 14% of the isolates (41). Another transporter, QacC (NCBI protein accession no. WP_000121134.1), which confers resistance to quaternary ammonium compounds, was present in a single isolate (Fig. 3E) (42, 43). These isolates were recovered during a period when sanitizers containing BC compounds were being used in the facility.

Stress survival islet 1 (SSI-1), which has been linked to tolerance toward acidic, bile, gastric, and salt stresses was present in 51% of the isolates and was observed in both lineages I and II (Fig. 3F) (44, 45). Only SL121 isolates (100%) harbored stress survival islet 2 (SSI-2) (data not shown). This island carries the *lin0464* and *lin0465* homolog genes, which are involved in survival under alkaline and oxidative stresses.

### Assessment of virulence factor genotypes across different sublineages.

The presence and integrity of *Listeria* pathogenicity islands 1 to 4 (LIPI-1 to LIPI-4) were investigated. The *prfA, plcA*, and *hly* genes present on LIPI-1 were present in all isolates except for L. monocytogenes CFS059 (SL31), where *prfA* was truncated. In 33% of the study isolates, *actA* was found to contain an in-frame internal deletion or truncation. Premature stop codons (PMSCs) within *inlA* were identified in 31% of the isolates representing SL9, SL31, SL121, and SL321 isolates (Fig. 3F). All the PMSCs identified were previously reported (46–48) (Table S2). Internal deletions within *inlB* were identified in 11% of the study isolates which all belonged to SL5.

10.1128/mSphere.00252-19.2 TABLE S2 Premature stop codons identified in *inlA* in this study. Download Table S2, DOCX file, 0.03 MB. Copyright © 2019 Hurley et al. 2019 Hurley et al. This content is distributed under the terms of the Creative Commons Attribution 4.0 International license.

LIPI-3 was present in nine lineage I isolates (SL1, SL3, SL6, SL213, SL218, and SL288) and a single lineage II isolate (SL288). A truncation was observed in the *llsY* gene carried on LIPI-3 in both L. monocytogenes CFS002 and CFS003 (both SL3). This gene encodes a putative posttranslational modification enzyme involved in oxazole production. The observed *llsY* alteration was previously reported in CC3 isolates (19). LIPI-2 was not observed in any of the study isolates, whereas L. monocytogenes CFS086 (SL213) was the only isolate containing LIPI-4 (Fig. 3F).

Previous studies have explored epidemiological data and the genomic traits associated with L. monocytogenes virulence (19, 49). L. monocytogenes containing PMSCs within *inlA* are putatively hypovirulent, whereas isolates with *actA* and *inlB* mutations are considered as having unknown virulence potential. Last, isolates belonging to clinically associated clones (including CC1, CC2, CC4, and CC6) are classified as putatively hypervirulent.

### SNP analysis and persistence.

Single nucleotide polymorphism (SNP) analyses were conducted on the isolate sequencing data for presumptive persistent CTs. SNP calls were made in comparison to a reference genome selected by leveraging all publicly available RefSeq genomes for *Listeria*. Briefly, a distance matrix of representative genomes for all nonredundant *Listeria* RefSeq genome clusters was generated using average nucleotide identity to determine the “closest” reference isolate to the study isolates from presumptive persistent CTs.

**FIG 4:**
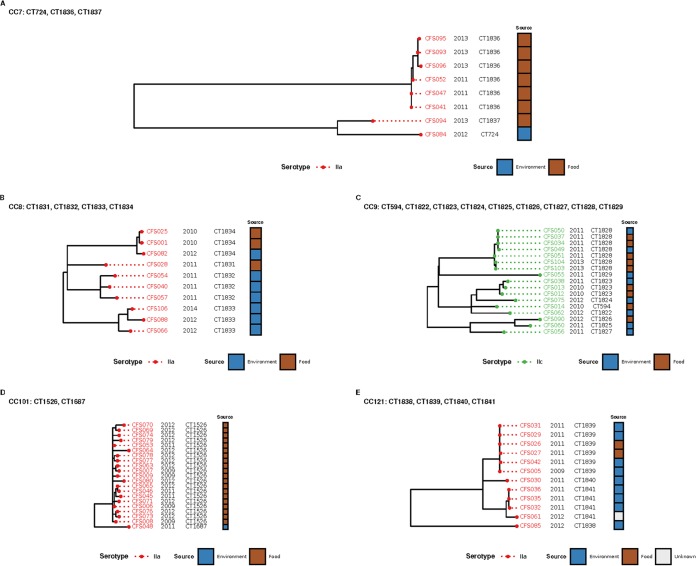
Approximate maximum likelihood phylogenetic trees based on SNP analyses of putatively persistent CTs. (A to E) Phylogenetic trees of CC7 with presumptive persistent CT1836 (A), CC8 with presumptive persistent CTs (CT1833 and CT1834) (B), CC9 with presumptive persistent CT1828 (C), CC101 with presumptive persistent CT1526 (D), and CC121 with presumptive persistent CT1839 (E).

The SNP analyses facilitated the identification of highly similar isolates, such as those belonging to CT1526, which were found to differ from the genome assembly GCF_003031955 by 1 to 17 SNPs. These isolates were cultured from raw food products (vegetables) over a 3-year period, suggesting that the raw ingredient supply was contaminated (Fig. 4A). Three isolates (CFS001, CFS025, and CFS082) were cultured from both environment and food sources and differed from the genome assembly GCF_001952775 by 0 to 5 SNPs (Fig. 4B). Similarly, SNPs identified in isolates within CT1828 differed from the genome assembly GCF_002557735 by only 0 to 5 SNPs and were cultured from both food and environmental sources, suggesting cross-contamination within the food processing facility (Fig. 4C). Isolates from CC101 were mostly CT1526 (*n *=* *21) and differed from the genome assembly GCF_001952775 by 1 to 17 SNPs (Fig. 4D). Last, a group of isolates in CT1839 differed from the genome assembly GCF_003030165 by 0 to 2 SNPs and were isolated less than 2 years apart from both food and environmental sources (Fig. 4E).

### Pathogenicity of selected L. monocytogenes with different virulence genotypes in a zebrafish embryo model of infection.

The pathogenicity of selected L. monocytogenes isolates with different virulence genotypes was characterized using a zebrafish embryo model of infection. Embryos aged 2 days postfertilization were separately microinjected into the caudal vein with wild-type L. monocytogenes EGD-e, four putatively hypovirulent isolates (L. monocytogenes CFS027 [SL121], CFS037 [SL9], CFS049 [SL9], and CFS059 [SL31]), one unknown virulence potential isolate (L. monocytogenes CFS002 [SL3]), and two putatively hypervirulent isolates (L. monocytogenes CFS086 [SL213]) and CFS087 [SL218]). The survival rate of the embryos was monitored for 72 h postinfection (hpi).

**FIG 5:**
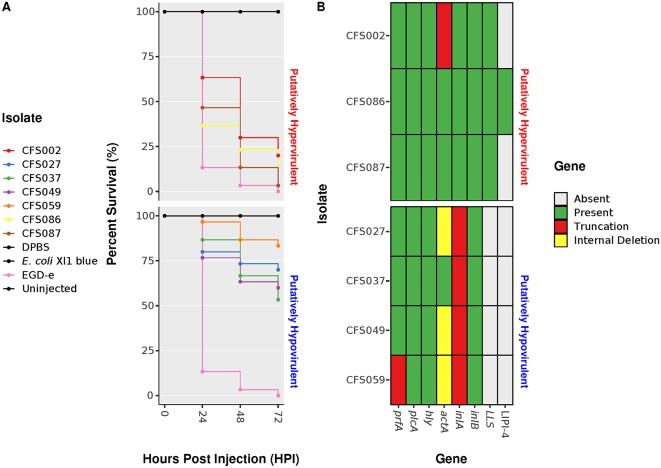
(A) Survival curves of zebrafish embryos injected with L. monocytogenes putatively hypovirulent isolates and L. monocytogenes unknown virulence potential and putatively hypervirulent isolates. Uninjected embryos and embryos injected with L. monocytogenes EGD-e, DPBS, and E. coli XL1-Blue were used as controls. (B) Heatmap showing the presence or absence of genes. Mutations, such as premature stop codons and internal deletions, are also shown.

Infection with wild-type L. monocytogenes EGD-e caused a rapid decrease in the survival rate of the embryos to 13% at 24 hpi, whereas embryos infected with putatively hypovirulent L. monocytogenes CFS027, CFS037, and CFS049 showed a significantly higher survival rate of 77 to 87% at 24 hpi relative to infection with strain EGD-e. Embryos infected with L. monocytogenes CFS037 or CFS049, which both harbor the PMSC type 11 in *inlA,* demonstrated similar survival curves. L. monocytogenes CFS059 harbors a truncation in *prfA*, the PMSC type 5 in *inlA* and an internal deletion in *actA*. Embryos infected with L. monocytogenes CFS059 exhibited the highest survival rate calculated at 97% at 24 hpi (Fig. 5A).

Embryos infected with L. monocytogenes putatively hypervirulent isolates CFS086 and CFS087 showed a rapid decrease in survival rate to 37 or 47%, respectively, at 24 hpi relative to unknown virulence potential and putatively hypovirulent isolates. Furthermore, embryos infected with CFS086 and CFS087 showed a lower survival rate compared to embryos infected with the unknown virulence potential isolate L. monocytogenes CFS002 at 48 and 72 hpi (Fig. 5A). Although L. monocytogenes CFS086 was the only study isolate harboring LIPI-4, embryos infected with CFS087 showed a lower survival rate at 48 hpi relative to CFS086 with survival rates at 72 hpi being the same (Fig. 5A and B).

## DISCUSSION

WGS is frequently used in public health settings for outbreak investigations (31, 33, 50–53), rather than for surveillance in food processing environments, to enhance the understanding of origin, cross-contamination, reservoir, and possible persistence of certain subpopulations along the food chain. In this study, WGS was applied as a surveillance tool for tracking L. monocytogenes in three related food processing environments over a 4-year period.

Previous studies reported high prevalence of ST9 L. monocytogenes in food processing facilities and in meat products from Spain and China (54, 55). In this study, 32% of the isolates originating from meat belonged to SL5, and 41% belonged to either SL7 or SL9. Furthermore, the most commonly identified SL among vegetable samples was SL101 at 69%.

Analysis of the data obtained from cgMLST genotyping allowed for the comparison of the core genome and the identification of highly similar isolates. The CT nomenclature facilitated the identification of international isolates with the same CTs. For example, a human isolate in Denmark was previously identified as CT550, whereas CT594 and CT724 were reported earlier in food matrices in France and England, respectively (35). In contrast, CT1819 and CT1850 were first encountered in this study and submitted to the BIGSdb-*Lm* database (34).

Six presumptive persistent CTs were selected for further SNP analyses, as these had been isolated at least three times with a minimum of 1 year between the first and the last isolations. The SNP analyses facilitated the identification of two different cross-contamination scenarios that can be hypothesized as follows. (i) The bacterium was introduced via the raw product, as closely related isolates were found in the raw product from three separate processing environments (cross-contamination events leading to the seeding of the food processing environment and the final product [e.g., isolates typed as CT1828]). (ii) The bacterium was introduced from the food processing environment to the final product or vice versa (e.g., isolates typed as CT1839).

Evidence of a contaminated raw ingredient supply was identified, as three CTs (CT1526, CT1828, and CT1836) were found repeatedly among the isolates from raw ingredients over 3, 2, and 2 years, differing by only 1 to 17, 0 to 5, and 1 to 6 SNPs, respectively. Isolates belonging to CT1836 were found repeatedly over a period of just under 3 years, differing by only 0 to 4 SNPs. Similarly, CT1828 isolates were recovered repeatedly over 2 years and differed by 0 to 5 SNPs. Isolates within CT1839 of SL121 differed by 0 to 2 SNPs, and all harbored transposon Tn*6188*, which encodes QacH, conferring potential resistance to benzalkonium chloride (BC), along with the SSI-2 stress survival operon (13, 37, 53). These two genomic traits may have conferred an advantage to survive under stress conditions which are routinely encountered in food processing environments (42, 43, 56).

BC tolerance genes (*bcrABC*, *emrC*, and *qacCH*) were identified in 73% of the presumptive persistent isolates. The most common BC tolerance gene identified was *emrC*, the efflux transporter associated with meningitis cases in the Netherlands (38). Interestingly, *emrC* was found in isolates of ST101 and not in ST6, as previously described by Kremer et al. (38). *bcrABC*, originally identified in L. monocytogenes isolated in Canada, was predominant among isolates from SL5 and SL9 (57). *emrE*, carried on the genomic island (LGI1) (42), was not found among the isolates tested, although it has been reported in isolates from Finland (58). Isolates that harbor BC tolerance genes may confer an advantage for survival under stress and in food processing settings, allowing the bacteria to persist in the environment (59, 60).

The *comK* gene, which has been hypothesized to be involved in virulence, biofilm formation, and persistence in food processing facilities (10, 36, 37), was found to be interrupted by the insertion of a prophage in 53% of the presumptive persistent CT isolates (44% of nonpersistent CT isolates). Although the percentage of putative persistent isolates carrying a prophage insertion within *comK* together with BC tolerance genes was considerable, isolates did not always harbor both genomic traits at the same time.

Examining the genomes of isolates in this study for virulence traits showed that the panel could be classified as putatively hypovirulent, unknown virulence potential, and putatively hypervirulent as previously described (19). L. monocytogenes containing PMSC mutations within *inlA* were considered putatively hypovirulent, as it has been demonstrated to be the main feature associated with loss of virulence, attenuating the ability of these bacteria to invade nonphagocytic cells (19, 61–63). In this study, putatively hypovirulent isolates were identified in SL9, SL31, SL121, and SL321, as these SLs harbor PMSCs within *inlA*, the most commonly identified mutation being PMSC type 6 (see Table S2 in the supplemental material). Furthermore, SL31 and SL121 harbored an in-frame internal deletion within *actA*, suggesting that these isolates may have reduced intracellular mobility.

L. monocytogenes isolates with unknown virulence potential represent intact virulence factors (for those factors studied, see Fig. 3E) as well as isolates harboring fewer mutations within virulence factors such as *actA* and *inlB*. Isolates from SL3, SL5, SL7, SL8, SL20, SL37, and SL101 are examples of unknown virulence potential. However, isolates from SL5 also harbored an in-frame internal deletion within *actA* and *inlB*.

Typically, clinically associated SLs are considered to be putatively hypervirulent (19, 29). These SLs show a low occurrence of mutations within the major virulence factors and possess a greater number of additional virulence factors, such as the LIPI-3 island that carries the gene encoding the hemolysin listeriolysin S, which contributes to the intracellular survival of L. monocytogenes in human polymorphonuclear neutrophils (64). Hypervirulent strains have also been shown to possess the recently described pathogenicity island LIPI-4, that confers hypervirulence by enhancing invasion of the CNS and placenta (19, 65). Fortunately, there was a low occurrence of putatively hypervirulent isolates in the food processing environments studied, despite isolates belonging to SL1, SL2, and SL6 being identified. Of note, the environmental isolate L. monocytogenes CFS086, from the newly identified sublineage SL213, harbored LIPI-4. LIPI-4 is highly prevalent in SL4, but it has also been identified in L. monocytogenes from SL87, SL88, SL315, SL569, and SL619 (34). Although the occurrence of putatively hypervirulent isolates was low, their association with food processing environments could have broad public health implications (41, 66).

The zebrafish embryo infection model was used to investigate the *in vivo* virulence potential of L. monocytogenes isolates with different virulence genotypes. Previous studies have used the zebrafish embryo infection model to investigate L. monocytogenes and its interaction with the host innate immune system (67, 68). Zebrafish embryo infections with putatively hypovirulent, unknown virulence potential, and putatively hypervirulent L. monocytogenes isolates showed different survival rates according to the virulence genotype of the isolate. Embryos infected with putatively hypervirulent and unknown virulence potential isolates showed a rapid decrease in survival rate after 24 hpi (37 to 47%), whereas embryos infected with putatively hypovirulent isolates required 72 hpi to decrease zebrafish embryo viability, resulting in a survival rate of 53 to 83% (Fig. 5A and B). In contrast, at 72 hpi, putatively hypervirulent isolates CFS086 and CFS087 exhibited 97% lethality, whereas the putatively hypovirulent isolate CFS037 showed a lethality of only 47%. These data support the approach used in this study to classify the potential virulence of L. monocytogenes isolates through WGS analyses, again highlighting the predictive advantage of this approach over traditional molecular subtyping approaches for a much broader range of genetic characteristics.

In food processing sites, surveillance and early detection are crucial to control L. monocytogenes occurrence and avoid cross-contamination. This study demonstrated the application of a WGS-based approach as a useful surveillance tool, in combination with a bioinformatic analysis targeting known biomarkers associated with persistence, antimicrobial resistance, as well as predicted hypovirulent and hypervirulent phenotypes. This approach facilitated the early identification and localization of presumptive persistent isolates within the food processing environment over a 4-year period. This information can be used to improve the management of the food processing environment, highlighting the need for adjustments to sanitation plans as required, while taking additional corrective actions when necessary. Furthermore, the development of robust and well-documented bioinformatic workflows, such as the CFSAN SNP pipeline, will enable deeper characterization of L. monocytogenes that will be of direct benefit to a food processing facility while maintaining consumer confidence through the protection of public health.

## MATERIALS AND METHODS

### Bacterial strains and growth conditions.

Over 4 years, from September 2009 to January 2014, routine surveillance was carried out in three meat and vegetable processing facilities on selected products in different stages of production in parallel with the food processing environments. A total of 100 L. monocytogenes isolates were cultured from environmental and food samples using ALOA One Day detection protocol (bioMérieux, Hampshire, UK), previously validated by ISO 16140:2003 and AFNOR/AES 10/3-09/00 (69). Among the 100 L. monocytogenes isolates, 35 were isolated from the environment and 64 from foods, with 1 having an unknown isolation source. The bacterial isolates used in this study are listed in Table S1 in the supplemental material.

The ALOA One Day detection protocol consisted of a two-step method, involving an enrichment step in half-Fraser broth for 24 h at 30°C and a detection step that requires plating 0.1 ml of the enriched culture on one ALOA plate and incubating for 24 h at 37°C. L. monocytogenes isolates were grown in brain heart infusion (BHI) (Oxoid, Hampshire, UK) broth with orbital shaking (200 rpm) at 37°C. Isolates were stored at −80°C in cryogenic vials (Thermo Fisher Scientific Inc., U.S.) containing 10% [vol/vol] glycerol (Sigma, Dublin, Ireland).

### Whole-genome sequencing.

Genomic DNA from all study isolates was purified using the Wizard genomic DNA purification kit (Promega, US). DNA libraries were prepared using the KAPA Low-Throughput Library Preparation kit with Standard PCR Amplification Module (Kapa Biosystems, Wilmington, MA), following the manufacturer’s instructions except for the following modifications. DNA (750 ng) was sheared using an M220 instrument (Covaris, Woburn, MA) in 50-μl screwcap microtube at 50 peak power, 20 duty factor, 20°C, 200 cycles per burst, and 25-s duration. Adapter-ligated fragments were size selected to 700 to 800 bp following Illumina protocols. Standard desalted TruSeq LT and PCR primers were obtained from Integrated DNA Technologies (Coralville, IA) and used at 0.375 μM and 0.5 μM final concentrations, respectively. PCR was reduced to four cycles. Libraries were quantified using the KAPA Library Quantification kit (Kapa Biosystems, Wilmington, MA), with 10-μl volume and 90-s annealing/extension PCR, before pooling and normalization to 4 nM. Pooled libraries were requantified by droplet digital PCR (ddPCR) on a QX200 system (Bio-Rad), using the Illumina TruSeq ddPCR Library Quantification kit following the manufacturer’s protocols, with an extended 2-min annealing/extension time. The libraries were sequenced using a V2 (2 × 250-bp paired-end) reagent kit on the MiSeq platform (Illumina) at a loading concentration of 13.5 pM, following the manufacturer’s protocols.

### Genome assembly and annotation.

The raw read quality was assessed with FastQC (version 0.11.8), and low-quality sequences were trimmed using Trimmomatic (version 0.39) (70). Trimmed, paired reads were *de novo* assembled using SPAdes (version 3.13.1) (71), and the resulting contigs were assessed with QUAST (version 5.0.2) (72), showing an average *N*_50_ of 663 kbp across these assemblies. Genome annotation was performed using Prokka (version 1.13.7) (73). Phage sequences were screened using PHASTER (74).

### Core genome MLST *in silico* subtyping.

The core genome MLST (cgMLST) analysis was performed using the BIGSdb-*Lm* platform (https://bigsdb.pasteur.fr/listeria) (34, 35). The cgMLST scheme consists of 1,748 highly conserved core loci representing 62% of coding regions from the L. monocytogenes EGD-e reference strain. This genotyping method defines cgMLST types (CTs) as groups of cgMLST profiles that differ by up to 7 allelic mismatches out of 1,748 loci and a sublineage (SL) as groups of cgMLST profiles that differ by up to 150 allelic mismatches out of 1,748 loci.

### PCR serogroup and MLST determination.

PCR serogrouping and seven-gene MLST scheme profiles were performed *in silico* for all the isolates studied using BLAST+ (version 2.9.0).

### Bioinformatic analyses of whole-genome sequencing data.

Genome assemblies were screened for the absence/presence of genes encoding antimicrobial resistance (AMR) using BLAST+ (version 2.9.0) and the ResFinder database (version 3.1.0) (75). Biocide resistance genes (*tetR*, *tnpABC*, *qacH*, *bcrABC*, *emrE*, *emrC*, and *qacC*) and *comK* were screened using the BLASTN algorithm with a minimum nucleotide identity and alignment length coverage of 80%. All genome assemblies were screened for the presence/absence of SSI-2, which includes *lin0464* and *lin0465* homologs using the BLASTN algorithm with a minimum nucleotide identity of 80%.

### Assessment of virulence factors.

The presence and integrity of virulence factors were assessed using L. monocytogenes EGD-e (GenBank accession no. NC_003210.1) as the reference genome for internalin A (*inlA*), internalin B (*inlB*), LIPI-1, and SSI-1. L. monocytogenes F2365 was used as the reference genome for LIPI-3 with the protein sequences LMOF2365_RS05570 to LMOF2365_RS05600, while L. monocytogenes LM9005581 was used as the reference for LIPI-4 with the protein sequences LM9005581_70009 to LM9005581_70014. Analysis was performed using the BLASTP algorithm with a minimum amino acid identity of 70%, allowing the identification of premature stop codons and internal deletions.

### SNP analysis.

SNP analyses were conducted using the CFSAN SNP pipeline (version 2.1.0) (76), mapping the raw reads of each genome to a reference assembly using Bowtie2 (version 2.3.5.1) (77). This strategy identified the variant call sites with VarScan (version 2.4.2) (78). SNP analyses were performed separately on presumptive persistent isolates from clonal complex CC7, CC8, CC9, CC101, and CC121. Calls were made in comparison to RefSeq genomes GCF_000568475.1 for CC7, GCF_001952775.1 for CC8, GCF_002557735.1 for CC9, GCF_003031955.1 for CC101, and GCF_003030165.1 for CC121. SNP matrices were used to construct phylogenetic trees using the approximate maximum likelihood approach in FastTree (version 2.1.11) (79).

### Maintenance of zebrafish cell lines and husbandry.

Zebrafish (Danio rerio) strains used in this study were *wik* lines. Adult fish were kept at a 14-h/10-h light/dark cycle at pH 7.5 and 27°C. Eggs were obtained from natural spawning between adult fish which were kept in pairs in individual breeding tanks. Embryos were raised in petri dishes containing E3 medium (5 mM NaCl, 0.17 mM KCl, 0.33 mM CaCl_2_, 0.33 mM MgSO_4_) supplemented with 0.3 μg/ml of methylene blue at 28°C. From 24 hours postfertilization (hpf), 0.003% 1-phenyl-2-thiourea was added to prevent melanin synthesis. Staging of embryos was performed by the method of Kimmel et al. (80).

### Microinjection experimental procedure.

Injections were performed using borosilicate glass microcapillary injection needles (Science Products catalog no. 1210332; 1-mm outer diameter, 0.78-mm inner diameter; Science Products, Hofheim, Germany) and a PV830 Pneumatic PicoPump (World Precision Instruments, Sarasota, FL, U.S.). The embryos (48 hpf) were manually dechorionated and anesthetized with 200 mg/liter buffered tricaine (Sigma catalog no. MS-222; Sigma-Aldrich, Buchs, Switzerland) prior to injection. Subsequently, the embryos were aligned on an agar plate and injected with 100 colony-forming units (CFU) (ranging from 90 to 142 CFU) in 1- to 2-nl volume of a bacterial suspension in Dulbecco’s phosphate-buffered saline (DPBS) (Sigma-Aldrich, Buchs, Switzerland) directly into the blood circulation (caudal vein). Prior to injection, the volume of the suspension was adjusted by injecting a droplet into mineral oil and measuring its approximate diameter over a micrometer scale bar. The following controls were included: infection with (pathogenic) L. monocytogenes EGD-e, infection with (apathogenic) Escherichia coli XL1-Blue, injections with DPBS, and noninjected embryos. The number of injected CFU was determined by injection of the same bacterial suspension used in the embryo infection experiments into a DPBS droplet on a BHI agar plate.

After injections, infected embryos were recovered in a petri dish with fresh E3 medium for 15 min. To monitor infection kinetics for survival assays, embryos were transferred into 24-well plates (one embryo per well) with 1 ml of E3 medium per well, incubated at 28°C, and observed for signs of disease and survival with a stereomicroscope twice a day. For survival assays after infection, the number of dead larvae was visually determined based on the absence of a heartbeat.

The number of dead larvae postinfection was determined at various time points visually based on the lack of a heartbeat. Experiments were conducted until 72 hpi. At the end of the infection experiments, embryos that were alive were euthanized with an overdose of 4 g/liter buffered tricaine. Usually, with the evaluation of distress and pain by behavioral observations, embryos were euthanized by prolonged immersion in overdose concentrations of tricaine solution (MS222; 200 to 300 mg/liter) and were left in the solution for at least 10 min until cessation of opercular movement. Since pain sensitivity has not developed at these earlier stages, before 96 to 168 hpf, this is not regarded as a painful technique. The maximum age reached by the embryos during experimentation was 5 days postfertilization (72 hpi) for which no license is required from the Swiss cantonal veterinary office since the embryos had not yet reached the free feeding stage. The methods applied were conducted following the approved guidelines.

### Data availability.

Accession numbers for raw sequencing data are in Table S1 in the supplemental material. Whole-genome sequencing data have been deposited at the Sequence Read Archive (SRA) under BioProject accession number PRJNA422580. Individual run accession numbers (SRR) for demultiplexed isolate data are listed in Table S1.
