# Supplementary material for: Whole-Genome Sequencing-Based Characterization of 100 Listeria monocytogenes Isolates Collected from Food Processing Environments over a Four-Year Period
Source: mSphere. 2019 Aug 7;4(4):e00252-19. doi: 10.1128/mSphere.00252-19 (PMC6686224; doi:10.1128/mSphere.00252-19)
Supplement: TABLE S2 [file mSphere.00252-19-st002.docx]

Table S2. Premature stop codons identified in *inlA* in this study.

| **PMSC mutation type^1^** | **Mutation position (nt)** | **Length truncated InlA (aa)** | **Strain number^¶^** | **Lineage** | **Sublineage** | **Source^2^** | **Reference** |
| --- | --- | --- | --- | --- | --- | --- | --- |
| 3 | 2100 (C → G) | 699 | FSL F2-516 | II | SL321 | EN (1) | Nightingale *et al.,* 2005 |
| 5 | 565 (C → T) | 188 | FSL R2-080 | II | SL31 | EN (1) | Van Stelten & Nightingale, 2008 |
| 6 | 1474 (C → T) | 491 | H1 | II | SL121 | EN (9), F (2), NA (1) | Olier *et al.,* 2003 |
| 11 | 2054 (G → A) | 684 | NV5 | II | SL9 | EN (2), F (6) | Rousseaux *et al.,* 2004 |
| 19 | 976 (G → T) | 325 | L3102 | II | SL9 | EN (2), F (1) | Gelbíčová *et al.,* 2015 |
| 29 | 1635 (deletion A) | 576 | SLCC2479 | II | SL9 | EN (3), F (3) | Moura *et al.,* 2017 |

^1^ As summarized by Gelbíčová et al., 2015.

^2^ EN, environment; F, food and NA, unknown.

**^¶^** denoted the isolate identifiers in the corresponding references
